# PTEN: An Emerging Potential Target for Therapeutic Intervention in Respiratory Diseases

**DOI:** 10.1155/2022/4512503

**Published:** 2022-06-30

**Authors:** Bangrong Cai, Liu Yang, Young Do Jung, Ying Zhang, Xinguang Liu, Peng Zhao, Jiansheng Li

**Affiliations:** ^1^Collaborative Innovation Center for Chinese Medicine and Respiratory Diseases Co-Constructed by Henan Province & Education Ministry of P.R. Henan Key Laboratory of Chinese Medicine for Respiratory Disease, Henan University of Chinese Medicine, China; ^2^Henan Research Center for Special Processing Technology of Chinese Medicine, School of Pharmacy, Henan University of Chinese Medicine, Zhengzhou 450046, China; ^3^Research Institute of Medical Sciences, Chonnam National University Medical School, Gwangju 501-190, Republic of Korea; ^4^Department of Cell Biology, School of Medicine, Jiangsu University, Zhenjiang, Jiangsu, China; ^5^Department of Respiratory Diseases, the First Affiliated Hospital of Henan University of Chinese Medicine, Zhengzhou 450000, China

## Abstract

Phosphatase and tensin homolog deleted on chromosome 10 (PTEN) is a potent tumor suppressor that regulates several key cellular processes, including proliferation, survival, genomic integrity, migration, and invasion, via PI3K-dependent and independent mechanisms. A subtle decrease in PTEN levels or catalytic activity is implicated not only in cancer but also in a wide spectrum of other diseases, including various respiratory diseases. A systemic overview of the advances in the molecular and cellular mechanisms of PTEN involved in the initiation and progression of respiratory diseases may offer novel targets for the development of effective therapeutics for the treatment of respiratory diseases. In the present review, we highlight the novel findings emerging from current research on the role of PTEN expression and regulation in airway pathological conditions such as asthma/allergic airway inflammation, pulmonary hypertension (PAH), chronic obstructive pulmonary disease (COPD), idiopathic pulmonary fibrosis (IPF), and other acute lung injuries (ALI). Moreover, we discuss the clinical implications of PTEN alteration and recently suggested therapeutic possibilities for restoration of PTEN expression and function in respiratory diseases.

## 1. Introduction

Chronic lung diseases such as asthma, pulmonary lung injury, chronic obstructive pulmonary disease (COPD), and idiopathic pulmonary fibrosis (IPF) are increasingly becoming an enormous global health concern and economic burden [1]. A growing body of evidence has shown that global exposure to polluted air, particles, toxins, and infectious microorganisms contributes to the development of these pulmonary diseases. Repeated exposure to these agents results in chronic airway inflammation and excessive lung tissue damage [2]. Despite great advances in the treatment of these diseases, there still remains a considerable unmet need for the development of safe and effective therapeutics. COPD is now the third leading cause of death worldwide, and no applicable drugs are available to reduce the mortality of COPD [3]. Although there are two therapeutic agents approved by FDA for IPF therapy, the current clinical therapy is unable to reverse the pathogenesis and only delays the progression of IPF [4].

**Figure 1:**
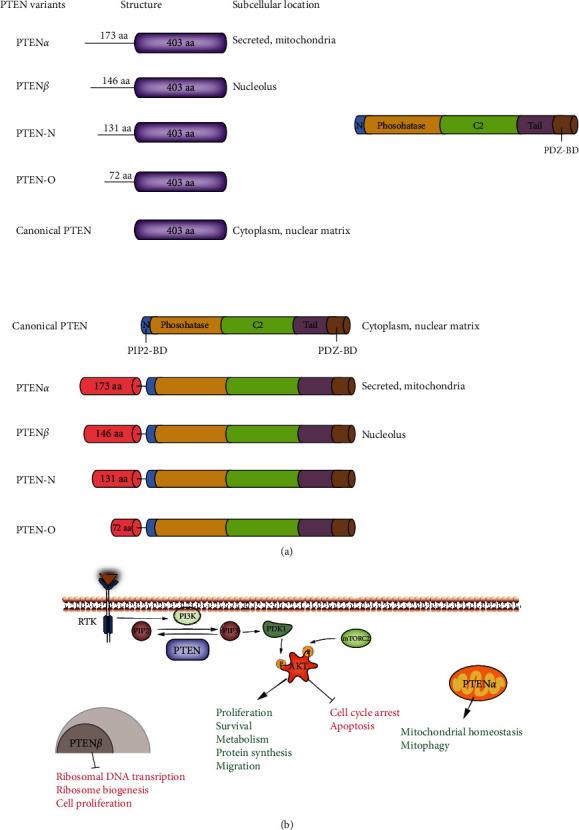
Molecular structure of PTEN (a) and cellular distribution of PTEN variants (b).

Identification of novel biomarkers and development of unique gene expression signatures are indispensable to improve early diagnosis and accurate prognosis, as well as to develop effective therapeutics. Accumulating evidence has demonstrated that phosphatase and tensin homolog deleted on chromosome 10 (PTEN) is one of the most important biomarkers that regulate multiple processes associated with the initiation and progression of various chronic lung diseases. It was initially identified as a potent tumor suppressor gene located on chromosome region 10q23, by three different groups in 1997 [5–7]. Canonical PTEN encodes a 403-amino acid peptide composed of five structural-functional domains. A short N-terminal PIP2 binding domain with phosphatase activity is responsible for dephosphorylation of phospholipids, a C2 domain for membrane targeting, a regulatory C-terminal tail containing multiple phosphorylation sites (Ser362, Thr366, Ser370, Ser380, Ser382, Thr382, Thr383, and Ser385), a PEST (Pro, Glu, Ser, and Thr) sequence, and a PDZ domain-binding motif involved in the regulation of PTEN activity/stability (Figure 1(a)). The cellular distribution of PTEN varies among tissues [8]. PTEN is primarily localized in the cytoplasm of most epithelial cells, such as those of the skin, breast, and prostate, whereas it is mostly distributed in the nuclei of neurons, fibroblasts, and thyroid cells [9, 10]. Moreover, differences in the subcellular distribution of PTEN have been observed in normal tissues and in several malignancies [9, 11].

A new isoform of PTEN with a 173-amino acid extension at the N-terminus, termed PTEN-long (PTEN-L) or PTEN*α*, was discovered in 2017; it differs from other isoforms in subcellular localization and function. PTEN*α* is secreted from cells and is directly exported into neighboring cells to inhibit cell proliferation [12], or it specifically localizes in the mitochondria to regulate cell metabolism [13]. PTEN*β*, another PTEN subfamily member, was identified by the same research group. PTEN*β* has an extension of 146 amino acid residues at the N-terminus and is mainly localized in the nucleolus and regulates preribosomal RNA synthesis [14] (Figure 1(b)). In addition, two other variants (PTEN-N and PTEN-O) have been reported; however, their specific roles remain unclear [15, 16]. Similar to PTEN, the abovementioned variants possess the ability to inhibit PI3K/Akt signaling [16].

PTEN is characterized as a dual-specificity lipid and protein phosphatase that can regulate the signal transduction pathways by both phosphatidylinositol-3,4,5-triphosphate (PIP3)-dependent, and PIP3 independent mechanisms. PIP3 is a potent secondary messenger that binds and activates proteins containing the pleckstrin homology domain, such as members of AKT family (AKT1, AKT2, and AKT3) and phosphoinositide-dependent protein kinase-1 (PDK-1). PTEN dephosphorylates PIP3, thereby inhibiting the activation of PI3K/Akt, NF-*κ*B, and the mammalian target of rapamycin (mTOR) signaling pathways, Akt/GSK-3*β* pathway, and the Akt/Wnt pathway. In addition, PTEN directly dephosphorylates focal adhesion kinase (FAK) and Src homology 2-containing protein (Shc). PTEN can autodephosphorylate itself on threonine residues within the regulatory C-terminal tail region [17], as well as dephosphorylate Ser, Tyr, and Thr residues of several other protein substrates such as FAK [18], IRS1 [19], CREB1 [20], and DVl2 [21]. In addition to lipid phosphatase activity, numerous studies have reported that PTEN exhibits nonenzymatic functions that are independent of PIP3 and PI3K/Akt pathway in both the cytoplasm and nucleolus. In the nucleolus, PTEN is involved in regulating diverse biological processes, including cell proliferation, transcription, and genomic stability. In the cytoplasm, PTEN was found to promote the activity of IP3R3 by competing with F-box/LRR-repeat protein 2 for IP3R3 binding in the cytosol, leading to Ca^2+^-mediated apoptosis [22]. Moreover, PTEN can stimulate proteasomal degradation of chromodomain-helicase-DNA-binding protein 1(CHD1) via *β*-TRCP E3 ubiquitin ligases to suppress CHD1-induced trimethyllysine-4 histone H3 modification, resulting in inhibition of transcription of oncogenic TNF-*α*/NF-*κ*B pathway [1].

Alterations in PTEN expression and activity are associated with the pathogenesis of cancer as well as a wide spectrum of other diseases. Loss of PTEN function frequently occurs due to a combination of genetic/epigenetic mechanisms, including various mutations, chromosomal deletions, and hypermethylation of gene promoters in various cancers. Moreover, PTEN expression and activity are subject to extremely complex regulation at the transcriptional, posttranscriptional (microRNAs and long noncoding RNAs), translational, and posttranslational levels such as oxidation, S-nitrosylation, acetylation, SUMOylation, ubiquitylation, and phosphorylation [23]. These posttranslational mechanisms impact the conformation, lipid phosphatase activity, subcellular compartmentalization, membrane localization, and stability of PTEN [23, 24]. In addition, PTEN interacts with other cellular proteins, thereby regulating their expression levels, activities, and stability [23, 25]. Alterations of PTEN expression and activity were comprehensively reviewed elsewhere [23, 25]. PTEN plays an important role in the suppression of lung cancer, and the effect of PTEN in lung cancer has been systematically reviewed [26]. Therefore, we only highlight the role of PTEN in diverse lung respiratory diseases in the present review. Additionally, the novel effective therapies by targeting PTEN for lung diseases are summarized.

## 2. PTEN in Chronic Lung Diseases

### 2.1. Asthma and Allergic Airway Diseases

Asthma is a chronic inflammatory respiratory disease characterized by airway hyperresponsiveness, airflow obstruction, mucus hypersecretion, and airway remodeling in response to inhaled allergens and nonspecific stimuli [27]. Growing evidence has revealed that significant reduction in the expression and function of PTEN contributes to the pathogenesis of asthma.

Some murine models of asthma induced by ovalbumin have been established to investigate the role of PTEN in the pathogenesis of asthma. Inflammation is the key pathogenesis of asthma. Lee et al. reported that the expression and enzymatic activity of PTEN were significantly reduced, which resulted in an increase in proinflammatory cytokines such as IL-4, IL-5, and eosinophil cationic protein in bronchoalveolar lavage fluids. Restoration of the PTEN expression could significantly reduce bronchial inflammation and airway hyperresponsiveness [28]. They further reported that PPAR-*γ* activation using rosiglitazone or pioglitazone agonists or adenovirus carrying PPAR-*γ* cDNA could upregulate the expression levels of PTEN, which inactivated PI3K/Akt pathway to inhibit bronchial inflammation and airway hyperresponsiveness in OVA-induced asthmatic mice [29]. Further, they revealed that restoration of PTEN expression to reduce the symptoms of asthma might be implicated in downregulation of VEGF [30]. In line with the abovementioned findings, Ni et al. reported that glucocorticoid dexamethasone treatment could transcriptionally upregulate the PTEN expression through inhibition of histone acetylation in asthmatic mice [31], suggesting that targeting PPAR-*γ* and histone deacetylase to restore the expression and function of PTEN is a promising strategy in the treatment of asthma.

Airway wall remodeling is another major pathological feature of asthma, and the increased proliferation and migration of airway smooth cells (ASMCs) play critical roles in these processes [32]. Since PTEN plays an important role in various cell processes, including cell growth, survival, proliferation, and migration, the repressed PTEN expression and activity promote the aggressive proliferation of ASMC that induces the airway remodeling. Numerous findings demonstrated that PTEN silencing promotes ASMC proliferation and induces airway remodeling. A study using a mouse model of OVA-induced asthma showed that loss of PTEN expression accelerates ASMC proliferation and forms a thick smooth muscle layer [33]. Lan et al. showed that the PTEN overexpression inhibits ASMC proliferation and migration by downregulating Akt and FAK signaling [34]. Furthermore, they proved that PTEN overexpression downregulated the levels of p-Akt and cyclin D1, and the enhanced expression of p21 suppresses proliferation and cell cycle arrest of ASMCs [35]. Additionally, Wu et al. demonstrated that the decreased expression of PTEN but increased CD38 expression was observed in TNF-*α*-induced ASMCs, whereas the overexpression of PTEN could remarkably downregulate CD38 expression, Ca^2+^ levels and phosphorylation of cyclic AMP response-element binding protein (CREB), and the proliferation and migration of ASMCs, suggesting that regulating PTEN/CD38/Ca2^+^/CREB signaling could restrict airway remodeling and inflammation in asthma [36]. Moreover, they illustrated that PTEN expression levels are inhibited by the increased expression of Notch1 via Hes1 in TNF-*α*-induced ASMCs, which facilitates ASMC proliferation, migration, and airway remodeling in asthma [37].

Several studies have demonstrated that the expression levels of PTEN are posttranscriptionally regulated by noncoding RNAs in various smooth muscle cells. Alexandrova et al. investigated the expression signature of small noncoding RNAs (sncRNAs) in asthmatic bronchial smooth muscle (BSM) cells. They found that 32 sncRNAs (26 miRNAs, five piRNAs, and one small nucleolar RNA) were aberrantly expressed in asthmatic patients, and nine miRNAs (miR-27b-3p, miR-92a-3p, miR-30a-5p, miR29a-3p, miR-186-5p, miR-103a-3p, miR-3182, miR-148b-3p, and miR-410-3p) were significantly upregulated in BSM cells from asthmatic patients compared to those in cells from healthy individuals. They identified 38 mRNAs as the major targets of these miRNAs through Ingenuity Pathway Analysis (IPA). Among them, PTEN mRNA was primarily targeted by miR-29a-3p and miR-92a-3p, resulting in abnormal activation of PTEN pathway in cells of asthmatic patients [38]. Hou et al. revealed that stimulation of high-mobility group box protein 1 (HMGB1) increases the expression levels of miR-19, which in turn downregulates PTEN expression, resulting in activation of PI3K/Akt pathway to accelerate ASMC proliferation [39]. Similarly, miR-620 targets PTEN and activates PI3K/Akt signaling to promote cell proliferation in TGF-*β*1-induced ASMCs [40]. Similarly, Lv et al. reported that TGF-*β*1 elevates miR-181a expression and suppresses PTEN expression by enhancing Akt/mTOR signaling pathway to promote ASMC proliferation, migration, and extracellular matrix (ECM) secretion [41]. The overexpression of miR-21 also represses the PTEN expression, thereby activating PI3K/Akt pathway and triggering the proliferation and migration of ASMCs [42]. Moreover, PTEN repression by miR-21 activates PI3K/Akt pathway, leading to decreased expression levels of nuclear histone deacetylase (HDAC) 2, thus enhancing inflammation in a severe steroid-insensitive asthma model [43]. Using a murine model of asthma and LPS-induced P815 mast cells, Zhou et al. suggested that PTEN levels are inhibited by the high expression of miR-221, accompanied by activation of p38 via phosphorylation, and NF-*κ*B signaling, which enhances IL-4 secretion [44]. Additionally, miR-21-5p also targets PTEN, resulting in the activation of signal transducer and activator of transcription 3 (STAT3) and PI3K/Akt/mTOR signaling, which aberrantly regulates airway wall remodeling in nonimmune immunoglobulin E- (IgE-) induced ASMCs [45]. It has been reported that long noncoding RNAs (lncRNAs) are involved in the regulation of the PTEN expression. LncRNA-CASC7 and lncRNA H19 upregulate the expression of PTEN by directly targeting miR-21 to inhibit the PI3K/Akt pathway, whereas downregulation of lncRNA-CASC7 and lncRNA H19 was observed in ASMCs from patients with severe asthma, suggesting that both these lncRNAs could be potential targets in the treatment of asthma [46, 47]. As aforementioned, dsyregulation of PTEN plays a key role in the aggressive proliferation of ASMCs, and restoration of PTEN might be an effective strategy to treat asthma. Several studies showed that bronchial epithelial cell injury is regulated by PTEN in asthma. Cui and Yang found that benzo [a] pyrene (Bap) treatment repressed PTEN and FAK expression and activates PI3K/Akt signaling in patients with asthma, which is believed to contribute to the bronchial epithelial injury caused by Bap-mediated ROS generation and cell apoptosis. Additionally, they showed that annexin A1 (ANXA1) protects against bronchial epithelial injury by increasing PTEN and FAK expression and inactivating PI3K/Akt pathway [48], suggesting that restoring PTEN expression might prevent bronchial epithelial apoptosis in asthma. The role of PTEN in regulation of asthma in detail was listed in Table 1.

### 2.2. Acute Lung Injury

Acute lung injury (ALI) is a severe clinical human disorder with high morbidity, caused by various conditions such as pneumonia, systemic inflammation, sepsis, major surgery, mechanical ventilation, or hyperoxia. The most important cell types that contribute to the pathogenesis of ALI are epithelial cells, macrophages, and neutrophils. Loss of PTEN has been observed in alveolar epithelial cells, macrophages, and neutrophils of ALI. Loss of PTEN function resulted in Akt/NF-*κ*B signaling pathway activation triggering lung inflammation-mediated injury. In addition, activation of PI3K/Akt signaling pathway plays an important role in the pathogensis of ALI.

Murine models of ALI induced by various stimuli are commonly used to investigate the role of PTEN in the pathogenesis of ALI. Fu et al. established a lipopolysaccharide- (LPS-) induced ALI mouse model to examine the effect of miRNA expression on PTEN transcription in lung tissues using miRNA microarray analysis. They found that the miR-92a expression was significantly increased in LPS-induced lung tissues; miR-92a suppressed the transcription of PTEN by binding to its 3′-UTR, and low levels of PTEN activated Akt/NF-*κ*B signaling pathway, resulting in lung inflammatory reaction in mice [49]. Another miRNA profile analysis by Lee et al. suggested that miR-21, which is upregulated in oleic acid- (OA-) induced ALI rats, directly repressed PTEN resulting in Akt pathway activation to induce ALI [50] suggesting that silencing miRNA to elevate PTEN expression might prevent ALI induced by various stimuli.

The function of epithelial cells is critical in wound healing in ALI. It was reported that reduction of PTEN levels disturbed the integrity of alveolar epithelial cells (AECs) due to the disassembly of tight junctions of AECs and destruction of the basement membrane via activation of PI3K/Akt signaling in ALI [51]. Conversely, other studies have suggested that PTEN repression has a protective effect on ALI via diverse mechanisms in AECs and lung epithelial cells. In addition, type II alveolar epithelial cell (AECII) apoptosis is an underlying mechanism involved in the pathogenesis of hyperoxia-induced acute lung injury (HALI). Qin et al. reported that elevated miR-21-5p levels in AEC II from lungs of HALI suppress PTEN expression, which in turn activates PI3K/Akt signaling, leading to decreased apoptosis of AEC II, thus ameliorating HALI [52]. Additionally, Wu et al. reported that miR-425 in the bone marrow mesenchymal stem cell- (BMSC-) derived exosomes (BMSC-Exos) inhibits PTEN levels, leading to activation of PI3K/Akt signaling, which attenuates HALI by promoting lung epithelial cell survival [53]. Tiozzo et al. highlighted that PTEN depletion extends tracheal epithelial progenitor cells and inhibits the differentiation of specialized epithelial cell types to confer resistance to lung injury [54]. Consistently, Knoell and colleagues found that PTEN inhibition activates PI3K/Akt pathway to improve wound closure and restore the integrity of lung epithelial monolayer for wound repair [55]. They further confirmed that PTEN repression could prevent ALI by improving epithelial cell tolerance in response to stress by activating PI3K/Akt pathway in a mouse model of OA-induced ALI [56]. They further demonstrated that loss of PTEN led to the activation of ERK and Akt signaling to enhance lung epithelial cell migration, thereby increasing wound healing following lung injury [57].

Inflammation is among the causative factors of lung tissue injury macrophage, alveolar macrophage and neutrophils play demonstrative role during the progression of ALI pathogenesis. A study in a mouse model of ALI by Zhou et al. suggested that HMGB1 induces PTEN expression, which in turn activates Foxo1 and TLR4 expression in alveolar macrophages, thereby triggering inflammation and causing ALI [58]. Furthermore, p38, along with its downstream target protein kinase D1 (PKD1), was reported to conversely regulate PTEN activity in neutrophils, thereby controlling migration of neutrophils in ALI [59].

Accumulation of alveolar edema fluid has been recognized as a common pathology of ALI, and alveolar fluid clearance (AFC) to remove edema fluid from alveolar spaces is critical for ALI recovery [60, 61]. Previous studies have highlighted that PTEN is closely associated with AFC, as it regulates the primary determinant, epithelial sodium channel (ENaC). In LPS-induced inflammatory lung injury, upregulation of miR-21 prevents PTEN expression, thereby resulting in PI3K/Akt activation and suppression of ENaC gamma (ENaC-*γ*) expression, which is the primary rate-limiting step in AFC. However, lipoxin A4 (LXA4) treatment enhances the ENaC*γ* expression and promotes AFC via miR-21/PTEN/Akt pathway to protect against LPS-induced ALI [62]. In contrast, Zhang et al. suggested that PTEN repression by miR-130b in BMSCs can promote the expression of ENaC alpha and gamma, which regulates severe pulmonary edema in ALI [63].

Sepsis is the leading cause of lung injury worldwide and occurs mainly due to the dysregulated host response to bacterial infection. In an experimental model of sepsis-induced lung injury, the results revealed that miRNA-23a improved sepsis-induced lung injury by repressing PTEN, leading to PI3K/Akt pathway activation and inhibition of p53 expression [64]. It has been reported that augmentation of PTEN in the leukocytes of both septic patients and mice enhances microbial clearance and inhibits lung damage and cytokine production. Nuclear localization of PTEN and its lipid phosphatase activity contribute to the increased production and maturation of miR-125b and miR-203b by directly regulating the translocation of miRNA-processing enzymes Drosha and DGCR8 in the nucleus, which limits the abundance of MyD88, resulting in the suppression of sepsis-induced lung injury [65]. Zhou et al. found that PTEN is activated by HMGB1, which is induced in endotoxin-stimulated macrophages during sepsis. Activated PTEN inhibits PI3K/PDK1/Akt signaling, which further suppresses *β*-catenin activity to modulate regulatory T cells, suggesting that HMGB1/PTEN/*β*-catenin signaling in the induction of regulatory T cells represents a novel therapeutic strategy in the treatment of sepsis-induced lung injury [66]. Intercellular interactions are involved in the regulation of lung injury progression, and miRNA-loaded exosomes play a key role in the transmission of signals between cells. AEC-derived exosomes induce pulmonary inflammation by activating alveolar macrophages via miR-92a-3p. The suppressed NF-*κ*B signaling is activated by miR-92a-3p in macrophages via downregulation of PTEN [67].

Acute respiratory distress syndrome (ARDS) is a severe form of ALI characterized by uncontrolled lung inflammation and lung epithelial and endothelial cell injury with enhanced pulmonary vascular permeability. Wang et al. suggested that PTEN is upregulated in the plasma and peripheral blood mononuclear cells of patients with ARDS, which is probably due to the increased expression of metastasis-associated lung adenocarcinoma transcript 1 (MALAT1), a multifunctional long noncoding RNA that interacts with miR-425 to induce cell apoptosis [68]. Pulmonary sarcoidosis is a systemic inflammatory disease, characterized by parenchymal granulomas with a silent, long-term evolution, and has progressively become the most common cause of sarcoidosis-associated death [69]. Several studies have investigated the role of PTEN in the development and progression of pulmonary sarcoidosis. The protein and mRNA expression levels of PTEN in bronchoalveolar lavage (BAL) cells of sarcoid patients were reported similar to those in the BAL cells of normal patients [70–72]. However, activation of the PI3K/Akt pathway was observed [70]. We presume that PI3K/Akt pathway activation probably occurs due to the reduced enzymatic activity of PTEN after posttranslational modification because PTEN is key suppressor of PI3K/Akt pathway, and the exact mechanism needs to be studied further. The detailed experimental information of PTEN involved in the development of lung injury is summarized in Table 2.

### 2.3. Chronic Obstructive Pulmonary Disease (COPD)

COPD is characterized by the progressive airway limitation, resulting in an abnormal inflammatory response in lung to noxious particles or gases. Cigarette smoke (CS) is considered as the primary cause of COPD, and CS exposure promotes oxidative stress that affects the expression level or function of PTEN by transcriptional and translational modification. In addition, several studies showed that genetic variation in PTEN was closely associated with COPD in substantial coal smoke exposure. Therefore, alterations in PTEN expression and function are closely associated with the development of COPD [73, 74].

In the cigarette smoke-exposed mouse model, a different alteration of PTEN was found. It has been reported that CS reduced PTEN level in macrophage cells, and the low level of PTEN leads to macrophage M2 polarization by PI3K/Akt activation in emphysematous mice [75], while Li et al. found that CSE exposure enhanced the stability of PTEN and promotes the expression of PI3K regulatory subunit PI3Kp85, which suppresses the phosphorylation of Akt through reducing the expression of protein arginine methyltransferase 6 (PRMT6) leading to CSE-induced epithelial cell apoptosis [76]. The difference of PTEN alteration suggested that PTEN regulated the progression of COPD through multiple mechanisms.

Numerous studies showed that reduced expression or function of PTEN was also found in COPD patients. Besiktepe et al. observed low expression levels of PTEN and HIF-1*α* in the lung samples of pulmonary emphysema (PE). The decreased PTEN expression may cause protease activation, decrease HIF-1*α* and lysyl oxidase (LOX), LOX-like protein 1 (LOXL1), LOXL2, and copper metabolism domain containing-1 (COMMD1), thereby contributing to the development of PE [77]. It has been reported that CS-induced oxidative stress inhibited PTEN expression [78], leading to increased PI3K/Akt signaling activity that promoted the production of proinflammatory mediators such as TGF-*β*, IL-6, CXCL8, CCL2, and CCL5, thereby COPD occurred [79]. Loss of PTEN function might be involved in COPD patients. We found that CSE exposure increased ROS generation that induced PTEN oxidation and impaired Trx-1 activity via dimerization, and the Trx-1 impairment delayed the reduction of PTEN and promoted PI3K/Akt signaling activation in BEAS-2B cells [80]. Additionally, Barnes et al. and Baker et al. illustrated that expression level of PTEN was downregulated by oxidative stress-induced miRNA-34 augmentation in BEAS-2B cells as well as peripheral lung samples from patients. PTEN reduction activated PI3K signaling to accelerate cellular senescence in COPD [81, 82].

Inflammation is recognized as an important driving force in the initiation, regulation, and development of COPD [83], and the repressed PTEN expression or function has been proved to induce inflammation in COPD. Numerous studies have shown that dysregulation of lncRNAs and miRNAs downregulated PTEN expression during the development of COPD. Shen et al. demonstrated that the low expression of lncRNA SHNG5 in COPD tissue was associated with increased inflammation and apoptosis. Downregulation of lncRNA SHNG5 increased the expression of miR-132, resulting in decreased expression of PTEN, which was probably due to miR-132, functions as a competing endogenous RNA for miRNAs [84]. Furthermore, Bozinovski and Anderson concluded that the acquired somatic mutations of PTEN in the epithelium of smokers are the major determinants of COPD. PTEN mutation intensified the inflammatory response by activating NF-*κ*B and AP-1 pathways in COPD [85, 86]. In addition, PTEN gene expression in COPD promoted MMP-9 expression in tumor-associated neutrophils by enhancing the STAT3-AP-1 interaction in bronchial epithelial cells, and loss of epithelial PTEN led to corticosteroid resistance [87].

Maintenance of lung epithelial integrity plays an important role in airway health, and epithelial barrier dysfunction contributes to the pathogenesis of COPD [88, 89]. The apical junctional complex (AJC) formed by tight and adherens junctions is crucial for epithelial barrier function [90]. Transcriptome analysis revealed that the expression levels of physiological AJC genes were globally reduced in the airway epithelia of smokers compared to those of nonsmokers. This was accompanied by a significant decrease in the expression of PTEN and its transcription factors, which was further downregulated in smokers with COPD, indicating that PTEN plays an important role in maintaining the integrity of epithelial junction barrier [78]. Endothelial barrier injury and inflammation are considered to be critical pathophysiological processes in CS-stimulated COPD. Inactivation of PTEN by phosphorylation plays an important role in endothelial dysfunction upon CSE exposure, particularly in the presence of inflammatory cytokines, through *β*-catenin-dependent gene regulation [91]. Among the various factors implicated in the development of COPD, proteases are specifically relevant to the pathophysiology of this disease. One such protease known to be upregulated in COPD is MMP-9, which regulates disease progression through augmentation of inflammation, extracellular matrix degradation, and neutrophil chemotaxis. A previous study suggested that loss of PTEN in COPD increases MMP-9 expression by enhancing STAT3-AP-1 interaction in bronchial epithelial cells [87]. PTEN plays an important role in preventing COPD, and alterations of PTEN are implicated in the development of COPD. Restoring the PTEN expression and activity is a promising therapeutic approach in COPD treatment. The effect of PTEN in the development and progression of COPD was summarized in Table 3.

### 2.4. Pulmonary Fibrosis

Pulmonary fibrosis is a progressive interstitial lung disease. Particularly, idiopathic pulmonary fibrosis (IPF) is characterized by excessive deposition of extracellular matrix molecules, such as collagen and elastin, with overactivation of fibroblasts/myofibroblasts. The pathogenesis of IPF remains largely unknown. Kulkarni et al. comprehensively investigated the target proteins and signaling pathways implicated in the pathogenesis of IPF in bleomycin-induced mice using a label-free LC-MS-based proteomics approach with systembiology. The results showed that mammalian target of rapamycin (mTOR) and extracellular signal-regulated kinase (ERK) are the primary regulators of pro- and antifibrotic responses. PI3K/Akt and Wnt signaling are key profibrotic pathways, whereas PTEN and natural killer cell signaling pathways are the most important antifibrotic pathways [92]. Numerous studies showed that the dysfunction and downregulation of PTEN were observed in IPF indicating that PTEN was involved in regulating the progression of lung fibrosis. The detailed information about the involvement of PTEN in lung fibrosis was listed in Table 4.

In the bleomycin-induced mice model of pulmonary fibrosis, myeloid PTEN-deficient mice exhibited the enhanced TGF-*β*1 activation and collagen deposition, decreased number of macrophages and T cells, and aberrant macrophage polarization with augmentation of various proinflammatory cytokines such as IL-6 and TNF-*α*. PTEN deficiency leadsto sustained PI3K activation in myeloid cells, thereby exacerbating IPF progression, suggesting that PTEN inhibits lung fibrosis via immunological mechanism [93]. The impairment of alveolar epithelial cells (AECs) contributes to IPF, and PTEN/PI3K/Akt pathway plays an important role in the maintenance and reconstitution of AEC integrity [94]. Miyoshi et al. reported that both patients with IPF and BLM-induced mice exhibited loss of AEC integrity and destruction of the basement membrane, which was accompanied by the decreased PTEN expression, indicating that PTEN plays a crucial role in the regulation of AEC integrity. PTEN deficiency intensified the disassembly of tight junctions of AECs and increased the abundance of epithelial-derived myofibroblasts and subsequent lung fibrosis [51]. In addition, Qui et al. reported that loss of PTEN promotes the senescence of AECs to induce lung fibrosis. Akt activation is considered to be the major mechanism underlying the reduced expression of PTEN [95]. Furthermore, they found that NF-*κ*B activation was involved in AEC senescence resulted from the reduced PTEN expression, and AEC senescence promoted collagen deposition of fibroblasts through the senescence-associated secretory phenotype [96]. Since cellular senescence is considered as an initial step contributing to lung fibrosis [81]. These findings suggest that therapeutic intervention of PTEN/Akt or PTEN/NF-*κ*B pathways might prevent the occurrence of IPF by suppressing the senescence process of AECs.

Aberrant lung fibroblast/myofibroblast activation and proliferation play important roles in fibrogenesis [97–99]. Decreased PTEN expression and phosphatase activity are frequently observed in fibroblasts or myofibroblasts from lung tissue of IPF patients [100, 101]. Geng et al. reported that the expression of USP13, a deubiquitylase that prevents PTEN ubiquitylation and degradation, is significantly reduced in lung tissues from IPF patients and in primary lung fibroblasts. The decreased expression of USP13 promoted PTEN ubiquitylation, leading to PTEN degradation, thereby contributing to fibroblast activation and pathogenesis of IPF [101]. Xie et al. found that PTEN expression is significantly reduced in Chinese patients with IPF; furthermore, a mechanistic study using TGF-*β*1-induced human embryonic lung fibroblasts (HFL-1) indicates that loss of PTEN resulted in activation of PI3K/Akt and TFG-*β*/Smad3 pathways [102]. In addition, PTEN is downregulated in fibroblasts insulted by various initiating factors such as bleomycin, paraquat, radiotherapy, smoking, and PM2.5, thereby causing lung fibrosis [103–105]. Additionally, radiotherapy induces miR-21-mediated PTEN repression, thus promoting EMT and development of lung fibrosis [105].

In an in vitro model of wound repair generated by human lung fibroblasts incorporated into type I collagen matrices, the expression levels of PTEN remain unchanged, whereas its lipid phosphatase activity is amplified in response to collagen matrix contraction. This led to impaired PI3K activity, resulting in reduced phosphorylation of Akt, thus sensitizing fibroblasts to collagen contraction-induced apoptosis [106]. Since IPF-derived fibroblasts were quite different from their normal counterparts, authors further investigated the differences between normal and IPF-derived fibroblasts cultured on polymerized type I collagen. The results showed that *β*1 integrin interacted with polymerized collagen and suppressed normal fibroblast proliferation through PI3K/Akt/S6K1 pathway owing to the enhanced lipid phosphatase activity of PTEN, whereas IPF-derived fibroblasts had low lipid phosphatase activity that resulted in high activity of PI3K/Akt pathway [99]. The extended study further showed that the expression levels as well as the lipid phosphatase activity of PTEN are necessary to induce myofibroblast differentiation, proliferation, *α*-smooth muscle actin (*α*-SMA) expression, and collagen production [97]. Moreover, the decreased phosphatase activity of PTEN was implicated in the low distribution of PTEN on cell membrane of IPF-derived fibroblasts [99]. Further study showed that low membrane-associated PTEN distribution probably resulted from the decreased expression of caveolin-1 that interacted with caveolin-1-binding sequence in PTEN and thus regulated the membrane levels of PTEN [98] suggesting that caveolin-1 is the key therapeutic target to inhibit IPF by regulating PTEN activity. Additionally, the low phosphatase activity of PTEN results in inhibition of downstream transcription factor forkhead box O3a (FoxO3a) via phosphorylation at Ser253, which reduces the expression levels of CDK inhibitor p27 and promoted cell cycle arrest in IPF-derived fibroblasts cultured on polymerized collagen [107]. Furthermore, they showed that the emerging apoptosis-resistant phenotype of IPF-derived fibroblasts is probably due to the aberrant regulation of PTEN/Akt axis, which inactivates FoxO3a, resulting in the downregulation of caveolin-1 suppressing the Fas expression in IPF-derived fibroblasts cultured on polymerized collagen [108]. The aberrantly altered PTEN/Akt axis may desensitize cell apoptosis induced by the polymerized collagen matrix through the inhibition of autophagy and enhancement of mTOR activity [109]. Another study reveals that FoxO3a expression at mRNA and protein levels is significantly suppressed, which caused low autophagic activity through transcriptional suppression of LC3B in IPF-derived fibroblasts cultured on a collagen-rich matrix, thereby promoting IPF progression [110] indicating that upregulation of FoxO3a and Fas to restore phosphatase activity of PTEN might effectively prevent IPF. Except for lung fibroblast activation, lung epithelial-mesenchymal transition (EMT) is one of the primary sources of myofibroblasts. Several studies showed that mechanical ventilation could promote EMT phenotypes of human primary AECIIs, and the augmentation of miR-19b inhibited the PTEN expression by posttranscriptional modification, which results in Akt activation [111].

Excessive proliferation of lung fibroblasts occurs during the early stages of IPF. It has been reported that lipopolysaccharide (LPS) treatment induces aberrant proliferation of mouse lung fibroblasts through activation of TLR4, which downregulates PTEN expression. The reduced PTEN expression results in PI3K/Akt pathway activation, thereby promoting fibroblast proliferation [112]. In addition to proliferation, excessive migration of fibroblasts also contributes to IPF progression, and loss of PTEN function promotes the migration and invasion of fibroblasts. White et al. reported that IPF-derived fibroblasts have low PTEN expression at mRNA and protein levels and decreased lipid phosphatase activity due to loss of alpha4beta1 expression, suggesting that PTEN plays an important role in suppressing fibroblast invasion and migration [113]. Furthermore, they reported that prostaglandin E2 (PGE2) inhibits fibroblast migration by targeting E-prostanoid (EP) 2 receptor, which leads to an increase in the lipid phosphatase activity of PTEN via dephosphorylation [114]. However, fibrotic fibroblasts are easily resistant to PGE2 owing to diminish the EP2 expression, which is attributed to hypermethylation of PGE receptor 2 gene promoter (PTGER2). Furthermore, PTGER2 methylation is probably regulated by the decreased PTEN expression and Akt signaling activation, which suggests that combination treatment using PGE2 and methylation inhibitors may be a potential therapeutic approach for the treatment of IPF [115]. Epigenetic regulation of PTEN gene expression was also found in silica-mediated lung fibrosis. Zhang et al. reported that promoter hypermethylation of PTEN in lung tissues from patients with silicosis might be implicated in the reduction of the PTEN protein expression [116].

The primary hallmark of pulmonary fibrosis is the formation of fibroblastic foci with excessive extracellular matrix (ECM) deposition, including collagen. Parapuram et al. reported that loss of PTEN expression results in excessive collagen deposition, which is closely related to constitutively increased expression of connective tissue growth factor (CCN2), a key regulator of tissue fibrosis [117]. Motohiro et.al demonstrated that TGF-*β*1 induced phosphorylation of PTEN at the C-terminus decreased its enzymatic activity and failed to inhibit ECM production in human fibroblasts, epithelial cells, and primary mouse lung fibroblasts. However, there was no effect on TGF-*β*1-induced *α*-smooth muscle actin expression in fibroblasts [118]. In the BLM-induced model of pulmonary fibrosis, myeloid PTEN-deficient mice exhibited enhanced TGF-*β*1 activation and collagen deposition, decreased number of macrophages and T cells, and aberrant macrophage polarization with augmentation of various proinflammatory cytokines such as IL-6 and TNF-*α*. PTEN deficiency led to sustained PI3K activation in myeloid cells, thereby exacerbating IPF, suggesting that PTEN inhibits lung fibrosis via immunological mechanisms [93]. These studies indicate that PTEN is implicated in PF progression by multiple mechanisms, and alterations in PTEN expression or function contribute to lung fibrogenesis.

### 2.5. Pulmonary Arterial Hypertension (PAH)

Pulmonary arterial hypertension (PAH), a severe and progressive disease characterized by increased pulmonary vascular resistance and pulmonary arterial pressure, is life-threatening. Shortness of breath, fainting episodes, and chest pain are the primary symptoms of PAH, and aberrant vascular remodeling resulting from the increased proliferation and reduced apoptosis of pulmonary arterial smooth muscle cells (PASMCs) is the primary features of PAH. PTEN has been reported to play a crucial role in the regulation of proliferation, differentiation, migration, and apoptosis of various cells. Recent studies have extensively investigated the involvement of PTEN in various murine models of PAH and hypoxia-induced human PASMCs. Dysregulation of PTEN leads to PI3K/Akt signaling activation, which increases the proliferation and migration of PASMCs associated with PAH. The detailed information about the involvement of PTEN in PAH was listed in Table 5.

Murine models of PAH were usually induced by monocrotaline, hypoxia condition, or monocrotaline combined with hypoxia. Numerous studies have reported that the low expression of PTEN was found in monocrotaline-induced PAH rat, and the decreased expression of PTEN was modulated by multiple mechanisms. Upregulation of miR-132 might repress PTEN expression to promote PASMC proliferation in PAH [119]. Augmentation of miR-17-5p reduced PTEN expression and cyclin-dependent kinase inhibitor 1 (p21) resulting in increased cell proliferation in hypoxia-induced PASMCs [120]. Oppositely, the increased expression of PTEN was also observed in monocrotaline-induced rats, which was associated with downregulation of miR-371b-5p expression. Increased expression of PTEN inactivated PI3K/Akt pathway, promoting apoptosis and reducing the proliferation of pulmonary arterial endothelial cells (PAECs) [121]. Additionally, low level of PTEN and phosphorylated-PTEN was attributed to ubiquitination-mediated degradation in monocrotaline-induced PAH rat, and decreased PTEN expression led to an increase in phosphorylated Akt, inactivation of cell-cycle regulatory proteins p53,p21, and p27, and accumulation of cyclin-D1 [122]. PTEN ubiquitination in monocrotaline-induced PAH rat was also modulated by elevation of NEDD4 [123]. In addition, the low expression of PTEN in human and monocrotaline-induced rat PAH was regulated by cAMP response element binding protein (CREB), a transcription factor that acts as a modulator of the vascular smooth muscle cell phenotype. Phosphorylation of CREB was inhibited with a concomitant decrease of PTEN, whereas a naturally occurring prostanoid prostaglandin E1 (PGE1) treatment could restore PTEN expression by elevating the phosphorylated levels of CREB indicating that PGE1 recruiting CREB/PTEN to inhibit PI3K/Akt signaling pathway can prevent the progression of PAH [124].

In the murine models of PAH induced by hypoxia, dysregulation of miRNAs contributes to the decrease in PTEN expression and function. Liu's group found that hypoxia significantly elevated the expression of miR-214 and miR-19 that targets PTEN to activate PI3K/Akt signaling pathway, thereby promoting cell proliferation of PASMCs [125, 126]. Low expression of PTEN was also implicated in repression of lncRNA MEG3 in hypoxia-induced PASMCs. MEG3 is a proliferation- and migration-inhibiting lncRNA, and it inhibits miR-21 to downregulate PTEN expression, thereby increasing proliferation and migration of PASMCs [127], suggesting that pharmacological intervention of miRNA or lncRNA to regulate PTEN level is an effective strategy for PAH treatment. Except for PTEN reduction in a variety of experimental PAH models, phosphorylation level of PTEN was increased in PAH lesions compared to normal lungs. In the mice model of specific deletion of PTEN in SMCs, they display a much severe PAH phenotype compare to wild type mice under hypoxia exposure for 4 weeks, indicating that PTEN is a key target for therapeutic intervention [128]. Additionally, the overexpression of TGF-*β*1 results in PTEN downregulation that activates PI3K/Akt signaling pathway to suppress the apoptosis of PASMCs [129]. In the model of PAH secondary to heart failure, high level of peroxynitrite might be responsible for PTEN downregulation, and peroxynitrite induces a significant decrease in the expression and activity of PTEN, which activated Akt pathway to promote proliferation of PASMCs and vascular remodeling, while reducing ROS production could restore PTEN expression and vascular remodeling in PAH secondary to heart failure, indicating that blunting ROS generation is a potential therapeutic approach [130].

### 2.6. PTEN as a Therapeutic Target

Given the central role of PTEN in a variety of chronic lung diseases, pharmacological modulation of PTEN expression and function at gene and protein levels is considered as a promising strategy in the treatment of chronic lung diseases [131]. Direct and indirect regulation of PTEN expression and activity with numerous modulators has been reported in the treatment of asthma, COPD, pulmonary fibrosis, and lung injury.

#### 2.6.1. Asthma

As mentioned above, chronic inflammatory respiratory and airway remodeling are the major pathological feature of asthma, and the mainstay therapy of asthma in clinic is anti-inflammatory and bronchodilator therapy. Natural products exhibit promising anti-inflammatory effect in diverse human diseases including asthma. Epigallo-catechin-3-gallate (EGCG) is a common polyphenol found abundantly in green tea and has diverse biological activities. Recently, it was reported that EGCG supplementation has a protective effect in ovalbumin- (OVA-) induced asthmatic mice. In term of mechanism, it inhibits EMT and migration via augmentation of PTEN expression in TGF-*β*1-induced 16HBE cells, thereby reducing inflammation and airway remodeling [132]. Resveratrol, another natural polyphenolic compound rich in red grapes, attenuates airway inflammation and remodeling in OVA-induced murine model of asthma through augmentation of PTEN mRNA and protein expression levels via SIRT1 activation [133]. A monoterpenoid, borneol, has been reported to delay the OVA-induced asthma progression in mice through downregulation of miR-26a and miR-142-3p that blocks PTEN transcription [134]. In addition, *α*- and *γ*-mangostin, major xanthones present in mangosteen fruit (*Garcinia mangostana* Linn), mitigate airway inflammation in OVA-induced allergic asthmatic mice by restoration of PTEN expression, which inactivates PI3K/Akt pathway and its downstream molecule NF-*κ*B, consequently reducing inflammatory cell recruitment into the airway and elevating the Th2 cytokine expression [135]. Lee's group reported that the Korean red ginseng extract and its component nepetin could attenuate Th2-derived cytokines, eosinophil infiltration, and airway remodeling in the OVA-induced allergic asthma model, and the mechanism is implicated in PPAR*γ* upregulation and PTEN-PI3K/Akt pathway inhibition by reducing the phosphorylation level of PTEN [136]. Although Korean ginseng extract and its component nepetin showed a promising antiasthmatic activity, no evidence for the active compounds and how to regulate PTEN phoshorylation was provided in this study. Pharmacological modulation of PTEN expression to inhibit PI3K/Akt pathway with natural products is an effective strategy in the treatment of asthma; however, whether those compounds directly regulate PTEN remains unknown. Dexamethasone is one of the most potent corticosteroids for asthmatic therapy in clinic, and it exerts the anti-inflammatory effect upon binding to the glucocorticoid receptor. It has been reported that dexamethasone inhibits inflammation partly by restoring PTEN expression and suppressing histone acetylase activity, which is usually decreased in the lung tissues of asthmatic mice [31] suggesting that dexamethasone regulates the PTEN expression level.

#### 2.6.2. Lung Fibrosis

Numerous studies exhibited that naturally occurring products have potential in the treatment of lung fibrosis by regulation of PTEN. Resveratrol inhibits the activation of TGF-*β*1-induced normal fibroblasts and IPF-derived fibroblasts, and the mechanism is probably related to upregulation of PTEN, which inhibits the phosphorylation of Akt and extracellular signal-regulated kinases (ERK1/2) [137]. Dasatinib, a platelet-derived growth factor receptor (PDGFR) and Src-kinase inhibitor, showed antifibrotic effect in bleomycin- (BLM-) induced mice and upregulation of PTEN expression after dasatinib treatment, which was decreased in BLM-treated mice [103]. It has been reported that paraquat (PQ) induces reduction of PPAR-*γ*, PTEN, TGF-*β*1, and *α*-SMA expression at both protein and mRNA levels, resulting in lung fibrosis in rats. The PPAR-*γ* agonist rosiglitazone attenuates PQ-induced pulmonary fibrosis by upregulating PTEN and decreasing the expression of TGF-*β*1 in a PPAR-*γ*-dependent manner [104]. Although restoring PTEN expression level by various natural products is accompanied by suppression of lung fibrosis, the exact mechanism remains unclear. The endogenous lipid mediator prostaglandin E2 (PGE2) showed increased apoptosis in normal and fibrotic lung fibroblasts against pulmonary fibrosis. This was associated with increased activity of PTEN, which inhibited the Akt signaling pathway. Furthermore, PGE2 reduced survivin expression and increased Fas expression in lung fibroblasts [138]. In addition, it inhibited fibroblast migration by restoring the lipid phosphatase activity of PTEN in an E-prostanoid (EP) 2 receptor-dependent manner [114]. The endogenous compound is nontoxic and further revealing the mechanism is necessary to find a potent lead compound. Berberine, an isoquinoline alkaloid, exhibited a marked protective effect against BLM-induced mouse pulmonary fibrosis in a gut-dependent manner; however, only oral administration was effective but not intravenous injection. The underlying molecular mechanism involves PPAR-*γ* activation that promotes the expression of HGF in colonic fibroblasts and increases HGF levels, which reaching the lung tissues through blood circulation to attenuate IPF. Moreover, berberine augments PTEN mRNA and protein expression levels in BML-induced mice [139]. Another study showed that, mechanistically, berberine attenuated lung fibrosis by suppressing the phosphorylation of Smad 2/3, augmenting Smad 7, and blocking FAK-dependent PI3K/Akt-mTOR signaling pathway [140]. Berberine is a multitarget drug and shows great potential in the treatment of lung fibrosis.

Silica exposure triggers lung inflammation and pulmonary fibrosis. Alveolar macrophages play a key role in the inflammatory response during silicosis. Coelonin, a dihydrophenanthrene compound isolated from *Bletilla striata*, inhibits inflammation in lipopolysaccharide- (LPS-) induced alveolar macrophages. The anti-inflammatory reaction of coelonin is probably associated with augmentation of PTEN expression and inhibition of its phosphorylation at Ser380 site, thereby inactivating PI3K/Akt signaling pathway, as well as inhibition of G1 phase cell cycle arrest by preventing p27^kip1^ degradation and proinflammatory cytokine gene expression through suppression of NF-*κ*B activation [141]. In addition, Kimura et al. found that exogenous administration of mutant PTEN at the C-terminus (S380A, T382A, T383A, and S385A) could restore the enzymatic activation of PTEN insulted by TGF-*β*1 resulting in inhibition of ECM overproduction in epithelial cells and fibroblasts in lung fibrosis [118].

#### 2.6.3. COPD

Mucus hypersecretion is a major pathology of COPD, cystic fibrosis (CF), and chronic bronchitis. FOXA2 is a key transcriptional regulator that maintains airway mucus homeostasis and is inhibited in airway diseases. Recently, it was reported that exendin-4, an analog of glucagon peptide-1 (GLP-1), could attenuate the production of excessive mucus by restoring the FOXA2 expression in COPD, CF-diseased cells, and mouse lungs infected by *P. aeruginosa.* It was found that exendin-4 triggered GLP-1R-dependent PKA and PPAR-*γ* activation, which in turn increased the expression of PTEN and PTP1B phosphatases. Both PTEN and PTP1B inactivated the key kinases STAT6 and EGFR via dephosphorylation, thereby restoring FOXA2 function and mucus homeostasis [142]. Curcumin, a natural polyphenol compound abundantly found in the rhizome of *Curcuma longa*, could suppress acute lung injury and inflammatory cytokines in rats with acute pulmonary embolism (APE). It probably inhibited the expression of Sp1, resulting in the downregulation of miR-21, which enhanced the posttranscriptional regulation of PTEN, subsequently inhibiting NF-*κ*B signaling pathway-mediated lung inflammation in rats with APE [143]. Cefminox, a dual agonist of the prostacyclin receptor and PPAR-*γ* identified by virtual screening, could attenuate hypoxia-induced pulmonary hypertension in rats. The mechanism is associated with PTEN upregulation, which suppresses activation of Akt/mTOR signaling pathway. Moreover, it could enhance the production of cyclic adenosine monophosphate (cAMP), suggesting that cefminox has a promising potential in the treatment of pulmonary hypertension, as it targets prostacyclin receptor and PPAR-*γ* [144]. Ivacaftor, approved by FDA for cystic fibrosis therapy, is a potentiator of cystic fibrosis transmembrane conductance regulator (CFTR). Riquelme and colleagues revealed that ivacaftor could potentiate CFTR activity by promoting the membrane distribution of PTEN and increasing its function via direct interaction with CFTR, thereby suppressing PI3K/Akt signaling to inhibit hyperinflammation in response to *P. aeruginosa* infection [145]. DNA methyltransferase inhibitor 5-Aza-2′-deoxycytidine (5-Aza-dC) has been reported to attenuate hypoxic pulmonary hypertension through demethylation of PTEN promoter [146]. Although numerous potential therapeutic agents were identified to be effective against COPD by modulating the expression and function of PTEN, there is still no drug reaching to clinical trial.

#### 2.6.4. Others

MicroRNAs (miRNAs) have shown great therapeutic potential in diverse lung diseases. For example, miR-486 protects against PM2.5-induced cytotoxicity in human lung alveolar epithelial A549 cells by inhibiting the expression of PTEN and FOXO1 [147]. It has also been reported that intratracheal administration of exosomes secreted by mesenchymal stromal cells (MSCs) ameliorates lung edema and dysfunction as well as the production of various proinflammatory cytokines in a mouse lung ischemia/reperfusion (I/R) model by transporting miR-21-5p. The *in vitro* study showed that MSC-secreted exosomes improved the endothelial cell apoptosis by suppressing both intrinsic and extrinsic apoptotic pathways through inhibition of PTEN and PDCD4 via miR-21-5p in hypoxia/reoxygenation condition [148]. In addition, the exosome miR-371b-5p derived from the human alveolar progenitor type II cell (ATIIC) line (A549) alleviates bleomycin-induced mouse lung injury. This event is mediated by negative regulation of PTEN expression, resulting in the phosphorylation of Akt and its downstream signaling molecules, such as GSK3*β* and FOXOs, to promote ATIIC cell proliferation and survival. This indicates that ATIIC-derived exosome miR-371b-5p could be a promising therapeutic candidate for augmenting ATIIC proliferation/survival and promoting the reepithelialization of injured alveolar cells in various incurable lung diseases [149].

Moreover, resveratrol can also protect against methamphetamine-induced high permeability and apoptosis of alveolar epithelial cells by reducing ROS levels and activating SIRT1, which leads to PTEN upregulation, thereby repressing Akt activation [150]. Resveratrol inhalation can slow down age-related degeneration of lung structure and function by maintaining the integrity of alveolar epithelial type 2 cells. Since resveratrol has been identified as an agonist of deacetylase, it may mainly augment the SIRT1 expression, which leads to the increased phosphorylation of Akt and Mdm2, thereby promoting p53 destabilization, decreased Bax expression, and inactivation of PTEN by phosphorylation [151]. The cannabinoid *Δ*^9^-tetrahydrocannabinol (THC), which is the active component of cannabis, was found to suppress pulmonary inflammation in mice induced by staphylococcal enterotoxin B (SEB) through downregulation of miRNA 17-92 cluster, particularly miRNA-18a that represses posttranscriptional regulation of *PTEN* gene. This indicates that the increased expression of PTEN inactivates PI3K/Akt signaling pathway, thereby reversing SEB-induced toxicity and death [152]. Modulation of PTEN with numerous modulators has been reported in the treatment of asthma, COPD, pulmonary fibrosis, and lung injury. Undoubtedly, restoring PTEN expression/function is a prime interest in the treatment of chronic lung diseases; however, it is extremely challenging to find a potent and direct PTEN modulators. The reported therapeutic agents as presented in Table 6 might indirectly regulate PTEN, and more studies are needed to fully clarify on the molecular mechanism.

## 3. Conclusion and Further Direction

In the present review, we highlighted the efforts made in the past few decades to investigate the role of PTEN in various chronic lung diseases such as asthma, COPD, IPF, PAH, and acute lung injury. PTEN is implicated in the regulation of various biological functions in lungs, ranging from inflammatory reactions, cell apoptosis, proliferation, differentiation, and so on. PTEN is abundantly expressed in various cell types of lung tissues such as macrophages, epithelial cells, endothelial cells, and smooth muscle cells. Both clinical and preclinical studies have shown that PTEN exerts a protective effect during the development and progression of diverse chronic respiratory diseases. Downregulation or inactivation of PTEN has been identified as the leading cause of almost all these diseases. The expression and activity of PTEN are subject to an extremely complex regulation at the transcriptional, posttranscriptional, translational, and posttranslational levels in various lung diseases. The specific mechanisms may vary in different types of diseases or different lung cell types. A variety of therapeutic agents such as noncoding RNAs, natural products, and clinical therapeutics aiming to restore PTEN expression/activity have showed great potential in the tested lung diseases *in vitro* and *in vivo*, but their exact mechanisms of action have not been fully elucidated. Further clinical trials are needed for these therapeutic agents. We conclude that PTEN plays a multifaceted role in the pathogenesis of lung disorders and is a promising therapeutic target for chronic lung diseases.

## Figures and Tables

**Table 1:** Biological functions of PTEN in the development of asthma.

Study type	Model/sample	Impact on PTEN	Additional signaling	Biological process	Ref.
In vivo	Female BALB/c mice/OVA-induced	Decreased PTEN expression and activity	Activated PI3K signaling	Increased bronchial inflammation and airway hyperresponsiveness in asthma	[28]
In vivo	Female BALB/c mice/OVA-induced	PTEN expression increased by PPAR-*γ*	Reduced PI3K activity	Inhibited allergen-induced bronchial inflammation	[29]
In vivo	Female C57BL/6 mice	Inhibited PTEN expression	Activated HIF-*α* and VEGF signaling	Increased inflammation and vascular permeability	[30]
In vivo/in vitro	Female BALB/c mice/OVA-induced; A549 lung epithelial cell line	PTEN expression increased by dexamethasone treatment	Histone acetylation inhibition	Dexamethasone treatment upregulated PTEN and exhibited anti-inflammatory effect in asthma	[31]
In vivo	Female BALB/c mice/OVA-induced	Decreased PTEN expression		Promoted ASMC proliferation and airway tissue remodeling	[33]
In vitro	Human airway smooth muscle cells (ASMCs)	Overexpression of PTEN	Downregulated Akt and FAK signaling activity	Inhibited ASMC proliferation and migration	[34]
In vitro	Human ASMCs	Overexpression of PTEN	Downregulated Akt signaling and cyclin D1 expression, upregulated p21 expression	Inhibited ASMC proliferation and induced cell cycle arrest in the G0/G1 phase	[35]
In vivo/in vitro	Female BALB/c mice; mice Airway smooth muscle cells (ASMCs)/ TNF-*α*	Decreased PTEN expression	Increased CD38-mediated Ca^2+^/CREB signaling	Promoted ASMC proliferation and airway tissue remodeling	[36]
In vitro	Mice airway smooth muscle cells (ASMCs)/TNF-*α*	Inhibited PTEN expression	Increased Notch1 expression	Facilitated ASMC proliferation and migration	[37]
In vivo/in vitro	Lung tissue specimens from asthma patients; bronchial smooth muscle (BSM) cells	Deregulated PTEN signaling	Increased miR-29a-3p and miR-92a-3p expression	Regulated cellular process in asthma	[38]
In vitro	Human ASMCs/HMGB1	Decreased PTEN expression	Activated the PI3K/Akt pathway and upregulated miR-19	Promoted ASMC proliferation and migration	[39]
In vitro	Human ASMCs/ TGF-*β*1	Decreased PTEN expression	Activated the PI3K/Akt pathway and upregulated miR-19	Induced ASMC proliferation and inhibited apoptosis	[40]
In vitro	Mice airway smooth muscle cells (ASMCs)/TGF-*β*1	Decreased PTEN expression	Upregulated miR-181a and activated the Akt/mTOR pathway	Promoted airway smooth muscle cell proliferation and airway remodeling	[41]
In vitro	Human ASMCs/miR-21 lentiviral vector	Decreased PTEN expression	Activated the PI3K/Akt pathway and upregulated miR-21	Promoted ASMC proliferation and migration	[42]
In vivo	Murine model of established allergic airway disease (AAD)	Inhibited PTEN expression	High levels of miR-21 enhanced the PI3K/Akt pathway and suppressed nuclear histone deacetylase (HDAC2)2 levels	Induced airway hyperresponsiveness in severe, steroid-insensitive asthma	[43]
In vivo/in vitro	Female BALB/c mice; P815 cells	Suppressed PTEN expression	Increased miR-221 activated p38 and NF-*κ*B signaling	Stimulated IL-4 secretion in mast cells	[44]
In vivo/in vitro	Human bronchial biopsies from asthma patients; human ASMCs	Downregulated PTEN expression	Activated STAT3 and miR-21-5p	Induced ASMC remodeling	[45]
In vitro	Human ASMCs	Suppressed PTEN expression	LncRNA-CASC7 levels were suppressed, and miR-21 levels were increased; the PI3K/Akt pathway was activated	Enhanced corticosteroid sensitivity in severe asthma	[46]
In vivo/in vitro	Serum samples from asthma patients; human ASMCs	Suppressed PTEN expression	LncRNA-H19 levels were suppressed, and miR-21 levels were increased; the PI3K/Akt pathway activated	Promoted ASMC proliferation and migration	[47]
In vitro	Human bronchial epithelial cell line (BEAS-2B)	PTEN expression was repressed by Bap treatment	Repressed FAK expression and activated the PI3K/Akt pathway	Induced bronchial epithelial cell apoptosis and cell injury	[48]

**Table 2:** Biological functions of PTEN in the development of lung injury.

Study type	Model/sample	Impact on PTEN	Additional signaling	Biological process	Ref.
In vivo/in vitro	Specific pathogen-free (SPF) male BALB/c mice; murine macrophage RAW264.7 cells/LPS	Inhibited PTEN expression	Enhanced the PI3K/Akt and NF-*κ*B pathways and increased miR-92a expression	Induced inflammation in LPS-induced ALI	[49]
In vivo	Male Sprague-Dawley rats/OA-induced	Decreased PTEN expression	Activated the PI3K/Akt pathway and increased miR-21 expression	Stimulated airway smooth muscle cell proliferation	[50]
In vivo/in vitro	Specific PTEN-deficient mice; lung epithelial cell line (BEAS-2B)/TGF-*β*	Reduction of PTEN expression	Akt hyperactivation	Controlled alveolar epithelial cell (AEC) integrity	[51]
In vivo/in vitro	Sprague-Dawley rats; type II alveolar epithelial cells (AEC II)/ hyperoxia induced	Decreased PTEN expression	Activated the PI3K/Akt pathway and increased miR-21-5p expression	Reduced AEC II apoptosis in hypoxia-induced ALI	[52]
In vivo/in vitro	SD rats; bone marrow mesenchymal stem cells/ hyperoxia-induced	Inhibited PTEN expression	Upregulated the PI3K/Akt pathway	Decreased TUNEL-positive cell number, induced cell viability, and reduced apoptosis	[53]
In vivo/in vitro	PTEN^Nkx2.1cre^ mice; PTEN^Nxk2.1-cre^ epithelial cells	PTEN depletion	Increased p-Akt and *β*-catenin activation	Expansion of lung epithelial progenitor cells leading to resistance to ALI	[54]
In vitro	Primary human upper airway epithelial cells (hUAECs), BEAS-2B, DU145, LNCaP, and PC3 cells	PTEN inhibition	Activated the PI3K/Akt pathway	Restored epithelial monolayer integrity for wound healing	[55]
In vivo	Adult C57BL/6 mice/OA-induced	PTEN inhibition	Activated the PI3K/Akt pathway	Enhanced epithelial cell tolerance to stress to mitigate ALI	[56]
In vitro	hUAECs and BEAS-2B cells/ mechanical scrap	PTEN inhibition	Activation of Akt and ERK signaling	Enhanced epithelial cell migration to improve wound healing	[57]
In vivo/in vitro	Male C57BL/6 mice; alveolar macrophage/ mechanical incision	Increased PTEN expression	Increased Foxo1 expression and NF-*κ*B activation	Activated TLR4-driven inflammatory response in ALI	[58]
In vivo/in vitro	p38 knockout mice; HL-60, RAW264.7 cells		Aberrant p38*δ*–PKD1 signaling	Induced neutrophil migration to inflammatory sites to cause inflammation in lung	[59]
In vivo/in vitro	Specific pathogen-free adult male SD rats; A549 cells/LPS	Inhibition of PTEN expression	Upregulated miR-21, activated PI3K/Akt signaling, reduced the expression of ENaC-*γ*	Regulated alveolar fluid clearance in ALI	[62]
In vivo/in vitro	C57 mice; alveolar type 2 epithelial (AT2) cells	Repressed PTEN expression	Activated PI3K/Akt signaling to increase *α*/*γ*-ENaC protein	Regulated alveolar fluid clearance in ALI	[63]
In vivo/in vitro	Wistar male rats; BEAS-2B cells/LPS	Repressed PTEN expression	Overexpressed miR-23a, activated PI3K/Akt signaling, and repressed p53 expression	Reduced lung apoptosis and attenuated lung injury	[64]
In vivo/in vitro	Female and male C57BL/6 mice; macrophages/cecal ligation and puncture (CLP)	Augmentation of PTEN	miR-125b and miR-203b induction and reduced MyD88 expression	Increased microbial clearance and prevented lung damage	[65]
In vivo/in vitro	Floxed *β*-catenin (*β*-catenin^flox^) mice; macrophages/LPS	Activated PTEN	Reduced PI3K/Akt and *β*-catenin activity, blocked macrophage TGF-*β* release, and decreased Foxp3+ Treg induction	Enhanced inflammatory response in sepsis-induced ALI	[66]
In vivo/in vitro	SD rats/ cecal ligation and puncture NR8383; RLE-6TN cells /LPS	Downregulation of PTEN via posttranscriptional modification	miR-92a-3p overexpression and NF-*κ*B signaling activation	Proinflammatory cytokine release	[67]
In vivo/in vitro	ARDS patients; human lung fibroblasts HFL-1 and A549 cells	Protected PTEN expression	Increased MALAT1 expression and decreased miR-425 expression	Promoted cell apoptosis in ARDS	[68]

**Table 3:** Biological functions of PTEN in the development of COPD.

Study type	Model/sample	Impact on PTEN	Additional signaling	Biological process	Ref.
In vivo/in vitro	Emphysema mouse model; bone marrow–derived macrophages (BMDMs); CSE-treated RAW264.7 and L929 cell lines	Decreased PTEN expression	Activated the PI3K/Akt pathway	Macrophage polarization toward the M2 phenotype in COPD	[75]
In vivo/in vitro	CS-exposed mouse model; BEAS-2B cells	Increased PTEN stability	Inhibited the PI3K/Akt pathway, decreased PRMT6 expression, promoted PI3Kp85 expression, and inhibited PDK1	Resulted in epithelial cell death in COPD	[76]
In vivo	Human lung tissue of COPD	Decreased PTEN expression	Activated HIF-1*α* signaling and MMP7/9	Decreased levels of oxidases (LOX, LOXL1, and LOXL2) caused abnormalities in elastic fiber biology	[77–79]
In vitro	CSE-exposed BEAS-2B cells	PTEN oxidation	Increased p-Akt level	Impaired Trx-1 activity	[80]
In vivo/in vitro	Lung tissues of patients with COPD; cells collected from patients undergoing lung resection surgery; BEAS-2B cells	Loss of PTEN expression	PI3K/mTOR signaling activation and SIRT1/6 inhibition	Caused cell senescence in COPD	[81, 82]
In vivo/in vitro	Human peripheral lung tissue; normal human bronchial epithelial cell line (16HEB)	Downregulation of PTEN	Decreased lncRNA SHNG5 sponge miR-132 expression	Regulated effects of CSE on cell proliferation, apoptosis, and inflammation	[84]
In vivo	Patients with COPD	Decreased PTEN expression	Enhanced the STAT3-AP-1 interaction	Increased MMP-9 expression to regulate airway remodeling	[87]
In vitro	Mouse cardiac endothelial cells (MCECs)	Low expression of PTEN	ROS/Src/EGFR-p38MAPK pathway	PTEN pathway related with AJC transcriptional reprograming to regulate epithelial barrier	[78, 90]
In vitro	Human nasal epithelial cell line (RPMI 2650)	Reduced PTEN activity	Increased TLR4/JNK/Bnip3 signaling	Activated mitophagy and induced mitochondrial dysfunction to cause epithelial cell apoptosis, proliferation arrest, and migration inhibition	[91]

**Table 4:** Biological functions of PTEN in the development of pulmonary fibrosis.

Study type	Model/sample	Impact on PTEN	Additional signaling	Biological process	Ref.
In vivo	Myeloid PTEN-deficient mice/bleomycin	Loss of PTEN expression	Sustained activation of PI3K pathway	Increased TGF-*β*1 activation, collagen deposition; reduced number of macrophages and T-cells	[93]
In vivo/in vitro	Human IPF lung tissue; IPF lung tissue; C57BL/6 and A549 cells/bleomycin	Loss of PTEN	P21WAF1, P16ink4, and SA-*β*-gal overexpression; NF-*κ*B and Akt activation	Alveolar epithelial cell senescence promotes lung fibrosis	[94, 95]
In vivo/in vitro	Human lung tissue; C57BL/6 embryonic mouse fibroblasts and 3T3 murine fibroblasts/TGF-*β*1; C57BL/6 mice/bleomycin	Diminished PTEN expression and phosphatase activity	Inhibition of PTEN activity in IPF-derived fibroblasts	*α*-SMA expression, cell proliferation, collagen production, and myofibroblast differentiation	[97]
In vivo/in vitro	Primary fibroblast cell lines from IPF and healthy lung/type I collagen–rich matrix; PTEN haploinsufficient and wild-type mice/bleomycin	High phosphatase activity in normal lung fibroblasts, but low activity in IPF-derived fibroblasts	Aberrant activation of the PI3K–Akt–S6K1 signaling pathway in IPF-derived fibroblasts	Enhanced the proliferation of primary lung fibroblasts	[99]
In vitro	Fibroblasts and myofibroblasts from patients with IPF; MRC-5 cells/H_2_O_2_	Loss of PTEN expression	Activated the TGF-*β*1 pathway and increased hyaluronan synthase 2 expression	Increased proliferation, apoptosis resistance, and migration/invasion activities	[100]
In vivo/in vitro	Human IPF lung tissue; MRC-5 cells/TGF-*β*1	PTEN ubiquitination and degradation	Downregulation of ubiquitin-specific peptidase 13 (USP13)	Enhanced proliferative, migratory, and invasive capacities of lung fibroblasts	[101]
In vivo/in vitro	Human IPF lung tissue; HFL-I cells/TGF-*β*1	Low expression of PTEN	Enhanced PI3K/Akt and TGF-*β*/Smad3 signaling	PTEN inhibited the proliferation and myofibroblast differentiation and promoted the apoptosis of fibroblasts	[102]
In vitro	Human lung fibroblasts CCL-210/mechanical stretch	Increased PTEN activity	Decreased Akt phosphorylation	Promoted fibroblast apoptosis	[106]
In vitro	Primary IPF-derived and normal fibroblasts/polymerized type I collagen	Low phosphatase activity	High Akt activity promoted the inactivation of FoxO3a and downregulation of p27 in IPF-derived fibroblasts	Facilitated fibroblast proliferation	[107]
In vitro	Primary control and IPF-derived lung fibroblasts/polymerized type I collagen	Low phosphatase activity	Inactivation of FoxO3a, which downregulated caveolin-1 and Fas expression	Apoptosis-resistant phenotype of IPF-derived fibroblasts	[108]
In vitro	Primary IPF-derived lung fibroblasts/polymerized type I collagen	Decreased phosphatase activity	Enhanced p-mTOR expression along with low expression of LC3-2 and FoxO3a	Suppressed autophagic activity	[109, 110]
In vivo/in vitro	Primary human alveolar epithelial type II (AEII) cells; small-airway epithelial cells/mechanical stretch	Downregulation of PTEN	miR-19a overexpression	Development of the EMT phenotype and lung fibrosis	[111]
In vitro	Murine embryonic fibroblasts/LPS	Low PTEN expression	Upregulation of TLR4 and PI3K/Akt pathway activation	Increased fibroblast proliferation	[112]
In vitro	Primary IPF-derived lung fibroblasts; normal human fetal lung fibroblasts (IMR-90)	Low PTEN expression and phosphatase activity	Loss of *α*4*β*1 signaling	Migratory/invasive phenotype of fibroblasts	[113]
In vitro	IMR-90 cells; murine embryonic fibroblasts/prostaglandin E2	Increased PTEN phosphatase activity by decreasing the phosphorylation of PTEN	E-prostanoid (EP) 2 receptor	Inhibited fibroblast migration	[115]
In vivo/in vitro	Human embryo lung fibroblasts/silica	Loss of PTEN expression due to hypermethylation of its promoter	MAPK and c-Jun methylation		[116]
In vitro	Deletion of PTEN or both PTEN and CCN2 in mouse fibroblasts	Loss of PTEN expression	Overproduction of collagen type I and connective tissue growth factor (CCN2)	Collagen deposition	[117]
In vitro	Epithelial H358 cells; normal human adult lung fibroblasts CC2512 and primary mouse lung fibroblasts /unphosphorylated PTEN/TGF-*β*1	Loss of PTEN enzymatic activity via phosphorylation of its C-terminus; retention of enzymatic activity in PTEN4A-treated cells	Suppression of *β*-catenin translocation by PTEN4A treatment	PTEN4A inhibits ECM production	[118]

**Table 5:** Biological function of PTEN in the development of PAH.

Study type	Model/sample	Impact on PTEN	Additional signaling	Biological process	Ref.
In vivo/in vitro	SD Rat/monocrotaline; human PASMCs/hypoxia	Low levels of PTEN	High expression of miR-132	Increased proliferation and migration of PASMCs	[119]
In vitro	Human PASMCs/hypoxia	Reduction of PTEN expression	High expression of miR-17-5p; down-regulation of p21	Aberrant proliferation and migration of PASMCs	[120]
In vivo/in vitro	SD rats/ monocrotaline; pulmonary arterial endothelial cells/MCT	Increases of PTEN expression	Downregulation of miR-371b-5p	Increased endothelial apoptosis	[121]
In vivo/in vitro	SD rats/monocrotaline, hypoxia; human PASMCs/hypoxia	Low expression of PTEN and reduced p-PTEN levels due to ubiquitination	Akt phosphorylation and inactivation of p53, p21, and p27	Increased proliferation of PASMCs	[122]
In vivo	Male SD rats/ monocrotaline, hypoxia	Low expression of PTEN due to its ubiquitination	Aberrant activation of PI3K/Akt signaling	Enhanced vascular remodeling	[123]
In vivo	Human PAH lung; PASMCs male SD rats/monocrotaline	Low levels of PTEN due to decreased p-CREB expression	PGE1 induced pCREB expression	Vascular remodeling and improved hemodynamics	[124]
In vivo/in vitro	Mice/hypoxia; human PASMCs/hypoxia	Decreased expression of PTEN	miR-19a overexpression enhanced PI3K/Akt signaling	Increased proliferation and migration of PASMCs	[126]
In vitro	Human PASMCs/hypoxia	Decreased expression of PTEN	Downregulation of lncRNA MEG3; over-expression of miR-21	Increased proliferation and migration of PASMCs	[127]
In vivo/in vitro	Human HAP; male SD rats/hypoxia	Decreased phosphatase activity or deletion of PTEN	Increased Akt phosphorylation	Increased pressure, extensive, pulmonary vascular remodeling, and increased macrophage accumulation	[128]
In vivo /in vitro	Adult female/male Wistar rats/hypoxia; human PASMCs/hypoxia	Low expression of PTEN	Activation of PI3K/Akt signaling	PASMCs resistant to apoptosis	[129]
In vivo/in vitro	Male SD rats/permanent ligation of left anterior descending; human PASMCs/hypoxia	Downregulation and decreased phosphatase activity of PTEN	Phosphorylation of Akt	Enhanced proliferative, vascular remodeling	[130]

**Table 6:** List of therapeutic drugs that modulate PTEN expression and activity in various lung diseases.

Diseases	Drug candidates	Models/samples	Effect on PTEN and target	Pharmacological effects	Ref.
Asthma	Resveratrol	Mouse/ovalbumin;16HBE cells	Restoration of PTEN expression and activation of SIRT1	Airway inflammation and airway remodeling	[132]
Asthma	Epigallo-catechin-3-gallate	Mouse/ovalbumin;16HBE cells/TGF-*β*1	Upregulation of PTEN and inhibition of PI3K/Akt	Suppresses inflammation and inflammatory cell infiltration; reduces airway remodeling by inhibiting EMT	[133]
Asthma	Borneol	Mouse/ovalbumin	Downregulation of miR-26a and miR-142-3p to upregulate PTEN expression	CD4+ T cell infiltration and proliferation	[134]
Asthma	*α*- and *γ*-mangostin	Mouse/ovalbumin	Upregulation of PTEN to suppress PI3K/Akt and NF-*κ*B signaling	Reduces inflammatory cell recruitment into the airway, airway hyperresponsiveness (AHR), and increased levels of Th2 cytokines	[135]
Asthma	Korean red ginseng and *Salvia plebeia* R.Br.	Mouse/ovalbumin	Downregulation of phosphorylated PTEN and Akt and upregulation of PPAR-*γ*	Reduces the levels of Th2 cytokines IL-4, IL-5, and IL-13 in BALF and splenocytes and downregulates the *IL-4, IL-13, IL-17, T-NF-α,* and *MUC5AC* genes	[136]
Fibrosis	Dasatinib	Mice/bleomycin	Upregulation of PTEN and inhibition of PDGFR-alpha; Src and c-Abl activation	Myofibroblast activation and collagen-1 accumulation	[105]
Fibrosis	Unphosphorylated PTEN	H358 cells, fibroblast CC2512 cells and mouse primary lung fibroblasts/TGF-*β*1	Restores the loss of PTEN activity	Reduces fibronectin expression and ECM production	[118]
Fibrosis	Resveratrol	Normal and IPF-derived lung fibroblasts/TGF-*β*1	Upregulation of PTEN and downregulation of p-ERK and Akt	Inhibits cell proliferation of both normal and IPF-derived fibroblasts, *α*-SMA expression, and intracellular collagen deposition	[137]
Fibrosis	Prostaglandin E2	Primary normal fetal lung fibroblasts IMR-90	Increases PTEN activity and decreases p-Akt; downregulates survivin expression; increases Fas expression	Fibroblast apoptosis	[138]
Fibrosis	Berberine	Mice/bleomycin	Upregulation of PTEN in the colon; activation of PPAR-*γ*	Promotes HGF expression in colonic fibroblasts, which arrive in the lungs to palliate IPF	[139]
Fibrosis	Berberine	Wistar rats/bleomycin	Amplifies PTEN expression to inhibit FAK and PI3K/Akt/mTOR signaling; inhibits p-Smad 2/3 and enhances Smad 7 expression	Inhibits fibrotic markers, *α*-SMA, fibronectin, and collagens I and III and reverses bleomycin-induced ultrastructural alterations in the lungs	[140]
Inflammation	Coelonin	Raw264.7 cells/LPS	Upregulation of PTEN and inhibition of PTEN phosphorylation, resulting in suppressed NF-*κ*B activation and p27^kip1^ degradation	Cell-cycle arrest in the G1 phase	[141]
Acute pulmonary embolism	Curcumin	Sprague–Dawley rats	Downregulation of miR-21 expression via inhibition of Sp1 to upregulate PTEN and impair the NF-*κ*B signaling pathway	Reduces mPAP and RVSP levels, W/D ratio, thrombus volume, and inflammatory factors	[143]
Pulmonary arterial hypertension	Cefminox	Primary rat pulmonary artery smooth muscle cells (PASMCs)/hypoxia	Upregulation of PTEN by inhibiting Akt/mTOR signaling and enhanced cAMP production	Inhibits growth of PASMCs as a dual agonist of prostacyclin receptor (IP) and PPAR-*γ*	[144]
Hypoxic pulmonary hypertension (HPH)	5-Aza-2′-deoxycytidine	Sprague–Dawley rats/hypoxia; PASMCs/hypoxia	Rescues the decreased PTEN expression by inhibiting hypermethylation	Proliferation, migration, and induction of apoptosis in PASMCs; pulmonary artery pressure and right ventricular hypertrophy index in HPH	[146]
Lung injury	miR-486 mimic	A549/PM2.5	Negative regulation of PTEN and FOXO1	Reduces cell apoptosis and ROS generation	[147]
Lung injury by ischemia/reperfusion	miR-21-5p	Mice/(I/R); primary murine pulmonary endothelial cells/H/R	miR-21-5p targeting PTEN and PDCD4	Reduces lung edema and dysfunction, M1 polarization of alveolar macrophages, and secretion of proinflammatory cytokines	[148]
Lung injury	miR-371b-5p	A549 cells	Targets PTEN to inhibit phosphorylation of Akt and its downstream substrates, GSK3*β* and FOXOs	Augments ATIIC survival/proliferation, thereby promoting reepithelialization of injured alveoli	[149]
Chronic lung injury	Resveratrol	Mouse/methamphetamine	Activation of Sirt1 to downregulate PTEN and upregulation of p-Akt	Reduces oxidative stress and reverses MA-induced higher permeability and apoptosis of alveolar epithelium	[150]
Lung injury	Inhaled resveratrol	terc−/−F2 C57Bl/6J mice	Inactivates p-PTEN and activates p-Akt and p-Mdm2 via activation of SIRT1	Maintaining AECII integrity and prevent deterioration of lung function	[151]
Lung injury	*Δ*9 Tetrahydrocannabinol	C3H/HeJ mice/Staphylococcal enterotoxin B	Posttranscriptional upregulation of PTEN via inhibition of miR-18a	Prevents SEB-induced mortality and alleviates symptoms of toxic shock	[152]

## Data Availability

The relevant data used to support the findings of this study are included within the article.
